# Laser-optic techniques for assessing blood microrheology in health and disease

**DOI:** 10.1007/s12551-026-01470-7

**Published:** 2026-10-17

**Authors:** Matvei Maksimov, Pavel Moldon, Danila Umerenkov, Kirill Korneev, Andrei Lugovtsov, Christian Wagner, Alexander Priezzhev

**Affiliations:** 1 Physics Department, Lomonosov Moscow State University, Moscow, 119991 Russia; 2 Dynamics of Fluids, Department of Experimental Physics, Saarland University, 66111 Saarbrücken, Germany; 3 Department of Physics and Materials Science, University of Luxembourg, Luxembourg, Luxembourg

**Keywords:** Erythrocytes, Optical tweezers, Laser aggregometry, Ektacytometry, RBC deformability, RBC aggregation

## Abstract

Blood microrheology and microcirculation is a vast field of research focusing on the properties of blood passing through lesser vessels and capillaries. In this part of the circulatory system, red blood cells (RBCs) determine the parameters of the flow, which impact the efficiency of oxygen transport and tissue supply. Various pathologies, such as cardiovascular diseases, diabetes mellitus, malaria and sickle cell disease alter parameters of RBCs severely impairing microcirculation. RBC abilities of reversible aggregation and deformation are among the main cellular properties ruling the hemorheology and microcirculation. In this review, we discuss five methods for blood microrheology and microcirculation in vitro studies: optical tweezers, flow chamber-coupled microscopy, laser aggregometry, laser diffractometry (ektacytometry) and flow cytometry. These techniques allow for assessing valuable cellular properties without significant damage to the cells during measurements. Some of the methods are used for studies on the scale of single cells, while others provide summarized parameters of thousands of cells. The possibilities of these techniques to study RBC aggregation and deformability are considered along with recent advances and current limitations.

## Optical tweezers

Several techniques can be implemented to trap and manipulate single cells, such as atomic force microscopy, micropipette aspiration, magnetic, acoustic and optical tweezers (OT), etc. (Wagner et al. 2013; Ozcelik et al. 2018; Depond et al. 2020; Hertz et al. 2023; Moreno et al. 2025; Korneev et al. 2026). While each of the methods has its advantages and limitations, optical tweezers are one of the best options for blood cell studies since it allows trapping live cells without mechanical contact and usually do not require complex sample preparation prior to measurements. Typically, optical trapping forces are in range of 0.1–100 pN. Deep learning techniques proved to be useful for optical tweezers measurements and should be applied if possible (Ciarlo et al. 2024).

The possibility of trapping micrometer-sized objects using only the forces of radiation pressure was first reported by A. Ashkin in 1970 (Ashkin 1970). Later, the technique was applied to trap single atoms and molecules, becoming widespread in the fields of quantum science and biophysics (Nieminen et al. 2007; Moffitt et al. 2008; Zhang and Liu 2008; Guo and Li 2013; Polimeno et al. 2018; Kaufman and Ni 2021; Huo et al. 2024; Stilgoe et al. 2024). The advancements in biological applications of optical tweezers led to the Nobel Prize in Physics awarded to A. Ashkin in 2018. Optical tweezers became one of the most noticeable tools in the field of microrheology and microcirculation as well (Zhu et al. 2020; Avsievich et al. 2020; Grigorev et al. 2023; Yang et al. 2026). Physical principles underlying this method and possible optical systems are described in detail elsewhere (Nieminen et al. 2007; Pesce et al. 2015, 2020). In this review, we focus on the variety of microrheological implementations of optical tweezers and discuss the latest achievements in this area.

### Membrane studies

Cell membrane conditions are one of the key determinants of cell deformability. The deformability of red blood cells (RBCs) is known to impact blood viscosity, especially at higher shear rates (Chien 1981; de Simone et al. 1990; Atwell et al. 2022; Ermolinskiy et al. 2024a). Thus, it is of interest to study single cell membrane properties at various pathologies and external stimuli.

One of the possible approaches is to study the deformability of vesicles with controlled composition instead of assessing RBC deformability (Zhang and Liu 2008). On the other hand, RBCs can be trapped and studied directly. RBC membrane viscoelastic properties were investigated in detail via optical tweezers (Zhu et al. 2020).

#### Single-trap studies

**Fig. 1:**
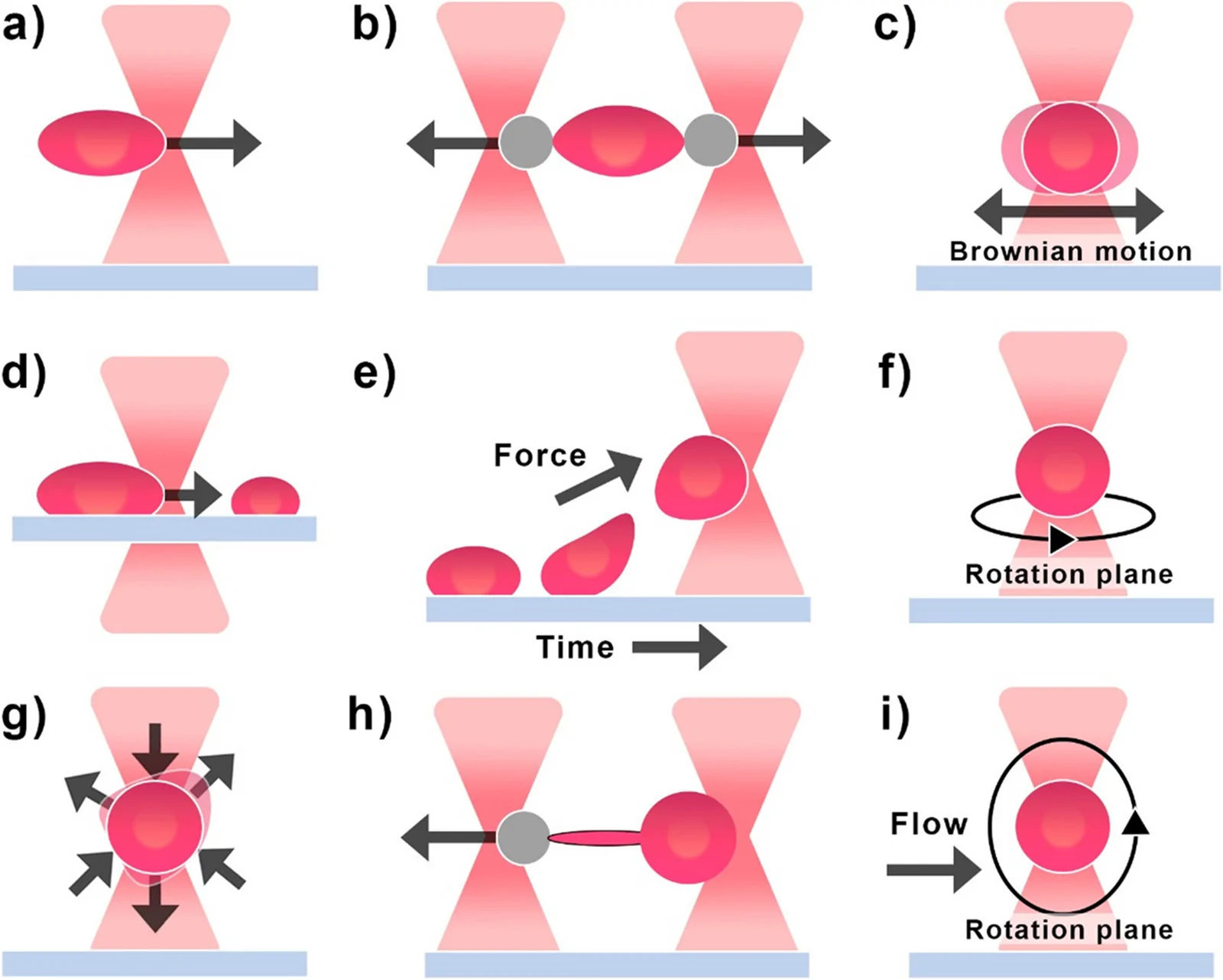
Possible techniques of assessing RBC deformability by optical tweezers: (**a**) trapping and dragging cell at various velocities, elongation is measured via microscope image, (**b**) stretching the cell by two trapped beads attached to the membrane, (**c**) Brownian motion of trapped cell is registered, its spectrum is further analyzed, (**d**) RBCs attached to the surface by one-point contact are “scanned” by moving optical trap, which subsequently deforms different cells, (**e**) RBC is trapped into optical trap, its folding dynamics is estimated, (**f**) RBC rotates in optical trap created by circularly polarized beam, (**g**) trap creates forces, which deform RBC membrane contour or excite its vibrations, (**h**) bead is attached to RBC membrane and pulled by a trap afterwards, at some point a tether is formed, which is further stretched and elongated, (**i**) trapped RBC in flow rotates in tank-treading motion

The simplest way to evaluate RBC deformability using OT includes trapping and dragging the cell at a certain speed with a following measurement of elongation index (the difference between major and minor axes of ellipse representing deformed cell divided by the sum of major and minor axes) (Fig. 1a) (Moura et al. 2015). Possible parameters also include apparent overall elasticity of the cell, which is calculated from the dependence of the cell length on the dragging velocity (Fontes et al. 2011). Recently, this technique was used to study the impact of nanoparticles on RBC deformability (Avsievich et al. 2023). Korobtsov et al. applied different approach to measuring RBC deformability: the cells are immobilized on the cuvette’s bottom, then the optical trap moves through the cuvette at certain velocity (Fig. 1d) (Korobtsov et al. 2012). Subsequently, cells get in the way of the trap and deform as it slides through them. Relative elongation was measured for each cell and deformability histograms were plotted for different osmolarities and sample preparation conditions. This approach is similar to flow chamber method (see flow chamber section). Trapped RBCs can also be studied in terms of Brownian motion (Fig. 1c) (Saraogi et al. 2010; Paul et al. 2017). The spectrum of Brownian fluctuations of trapped RBC is registered and the corner frequency characterizing it is calculated. The measurements were made at various trapping forces and it was shown that both corner frequency and its dependence on laser power significantly differ for malaria-infected cells compared to normal due to their increased rigidity. Optical tweezers were combined with optical confocal microscopy to excite membrane vibrations and study its viscoelastic properties (Fig. 1g) (Sirotin et al. 2020). Recent research demonstrates possibility of local membrane deformation by the complex application of holographic optical tweezers forces (Caselli et al. 2023).

A common optical tweezers technique for cell membrane studies involves trapping of beads (silica, polystyrene and others) and its attachment to the cellular membrane (Fig. 1b) (Yadav and Savopol 2024). Then, the cell can be manually deformed to measure its Young’s modulus and other parameters. Another possible technique implies direct RBC trapping by two optical traps and usage of another trap to deform central area of the cell (Bronkhorst et al. 1995).

#### Elongation using multiple traps

Single RBC is attached either to a movable bead and a static surface or to two beads, one of which is static, while the other is pulled by an optical trap. The possible measured parameters include shear modulus (ratio between applied shear stress and strain) and relaxation time (time required for RBC to restore its original shape), apparent membrane viscosity, zeta potential, etc. (Mills et al. 2004; Fontes et al. 2008; Song et al. 2017; Vos et al. 2024). Review by Zhang et al. summarizes the results acquired from 1995 to 2007 (Zhang and Liu 2008). Briefly, the elder RBC subpopulation is characterized by longer relaxation times compared to both the younger subpopulation and the total RBC population; shear modulus ranged from 2 to 200 μN m^−1^ depending on the setup and cell modifications; forces applied ranged from 0 to approximately 200 pN. RBC deformability was shown to depend significantly on the temperature, which limits in vitro measurements at room temperature. In case of malaria, RBC shear modulus was shown to increase tenfold in parasite-infected RBCs, the values ranged from approximately 5 to 100 μN m^−1^ in normal and infected cells, correspondingly (Suresh et al. 2005).

Furthermore, RBC deformability was studied by this technique at diabetes mellitus, the cells were trapped directly by two optical traps without using beads (Agrawal et al. 2016). The acquired deformability parameter was significantly lower both for diabetes mellitus and diabetic retinopathy patients compared to control group. Newest results demonstrate that hydroxyurea treatment of sickle cell RBCs improves their deformability (Mohi et al. 2021a).

Oscillating optical traps were shown to be an effective tool for RBC deformability estimation (Lyubin 2012). For higher stretching forces one may consider the implementation of so-called “tug-of-war” optical tweezers (Liang et al. 2019). Basically, spatial light modulator generates two diverging elongated beams, which are applied to a single RBC. The parameters (distance between traps, etc.) are regulated to stretch the cell and, for example, the axial diameter is measured. The results acquired demonstrate the dependence of the axial diameter on laser power and osmolarity.

#### Tether formation

Tether formation by optical tweezers for membrane characterization is widely applied to different cell types (Dai and Sheetz 1999; Huang et al. 2003; Nussenzveig 2018). Although this approach has been rarely used for RBCs, several studies are now available. Cardenas et al. indirectly observed RBC membrane tethers generated by optical trapping; however, tether mechanics and membrane properties were not quantified (Cardenas et al. 2011). More recently, Gironella-Torrent et al. employed optical tweezers to investigate the viscoelastic response of RBCs during tether extrusion and relaxation (Gironella-Torrent et al. 2024). Tether formation occurred at forces of approximately 30 pN and was accompanied by a marked decrease in the apparent cell stiffness and increased hysteresis of the force–extension curves. On the other hand, RBC tethers were studied intensively earlier via micropipette aspiration and flow chamber methods (Hochmuth et al. 1973; Waugh and Bauserman 1995; Hochmuth and Marcus 2002; Borghi and Brochard-Wyart 2007; Asaro and Cabrales 2021). The main drawback of these studies is the inaccuracy of forces applied to the cell.

Common technique of tether formation by optical tweezers includes trapping a bead (silica, etc.), adhering it to the surface of an immobilized cell (for example, via another optical trap or surface adhesion) and stretching the membrane by the bead (Fig. 1h). At a certain point a tether is formed, which can be further elongated to some extent. Tether parameters can be measured to evaluate cellular membrane properties. For example, optical tweezers can be used to form a tether between a leucocyte and micrometer-sized polystyrene bead as well as other cell types (Guo et al. 2003; Pontes et al. 2013; Guo and Li 2013). The force applied to the tether, the surface membrane tension and the bending modulus are measured. The force applied to the tether is determined by the displacement of the bead in the trap (the steady-state force is implied). This force increases with an increase in tether diameter and density (Guo et al. 2003). Membrane tension is the force applied to the tether divided by tether radius (and, also, divided by a constant of 4 π). Bending modulus is the force applied to the tether times tether radius (and divided by a constant of 2 π). Another possible set of measured parameters includes the tether length, plateau force and escape force (Titushkin and Cho 2006). If the tether is pulled at constant velocity, the force applied to it fluctuates around some level until a certain point, at which it increases drastically and, finally, the bead escapes the trap and returns to its original position. Plateau force indicates the level at which the force fluctuates at the initial pulling phase, escape force is determined as the maximum force, at which the bead escapes the trap, and the tether length is the maximum value of the tether length.

Tether formation and characterization appears to be a perspective technique for assessing RBC membrane properties. There seems to be no obvious limitations on the application of this method to this particular cell type, thus, further studies may provide unique insights into RBC membrane deformability both in health and disease.

#### Rotation and folding dynamics

RBCs fold when trapped by optical tweezers, the folding speed depending on laser power (Fig. 1e) (Ghosh et al. 2006; Spyratou et al. 2011; Mohi et al. 2021b). It also depends on the cell deformability, which allowed Bambardekar et al. to introduce folding-unfolding technique to estimate cellular properties at malaria disease (Bambardekar et al. 2008). The folding time is measured when RBC is introduced into the optical trap, typically ranging in milliseconds. The unfolding time is measured when the trapped cell is released and typically ranges in seconds. Both times were shown to correlate with the extent of the disease. Mohi et al. measured the folding times for sickle RBCs (Mohi et al. 2021b). This study introduced another possible parameter — RBC folding angle. It was found that folding and rotational parameters differ significantly in sickle cell disease group compared to healthy individuals. Thus, the folding dynamics provide insights into cellular deformability and are yet to be investigated at other RBC conditions.

Basu et al. demonstrated the possibility of observing tank-treading motion (specific rotational movement propelling cells to the center of the flow) of RBCs trapped by optical tweezers (Fig. 1i) (Basu et al. 2011). The main measured parameter is the frequency of this rotational movement. Experiments at various temperatures, osmolarities and external viscosities demonstrated that the frequency increases both with an increase in temperature and osmolarity. External viscosity was shown to have negligible effect on tank-treading frequency. This means that the frequency depends on such cellular properties as its internal viscosity and membrane rigidness. Both these factors significantly impact cell deformability, thus, the proposed technique of measuring tank-treading frequency via optical trap can be applied for assessing deformability of RBCs at different conditions.

Circularly polarized laser beam can be applied to trap and rotate single RBCs with even mass distribution (Fig. 1f). Malaria-parasite infected RBCs and other cells with uneven distribution of density can be rotated by both linearly and circularly polarized beams. Rotational speed was shown to depend on RBC properties and alters at such pathologies as malaria (Dharmadhikari et al. 2004). Alongside with folding time and tank-treading frequency rotational speed could also be used as a cell deformability parameter.

### RBC aggregation forces measurements

RBC aggregation impacts blood microrheology significantly and is known to increase at various pathologies, such as sickle cell disease, diabetes mellitus, cardiovascular diseases (Connes et al. 2016; Lugovtsov et al. 2019). RBC reversible aggregation should be distinguished from other intercellular interactions, for example occurring during blood coagulation (Yang and Li 2010). First attempts of RBC aggregation mechanisms investigation by optical tweezers were attempted by Bronkhorst et al. in 1996; however, the intercellular forces were not measured (Bronkhorst et al. 1997). Still, it was demonstrated that aggregating cells form tethers between them and that intercellular forces increase with aggregating time. To our knowledge, investigation of RBC aggregation forces by optical tweezers technique is limited to reports by five scientific groups, four of which conducted joint studies.

The group of Cesar applied optical tweezers to study RBC aggregation in terms of RBC pair membrane viscosity (Fontes et al. 2011; Fernandes et al. 2013). The group of Meglinski and Myllylä used optical tweezers to trap RBC aggregates (Kauppila et al. 2011), studied the impact of RBC interaction time on aggregation and disaggregation forces highlighting differences in mechanisms of these two processes (Zhu et al. 2019b). RBC aggregation in dextran solutions and autologous plasma was compared (Avsievich et al. 2018). RBC interaction energy was studied by optical tweezers and the effect of continuous laser irradiation on RBC disaggregation was shown (Zhu et al. 2019a, 2022), also, the interaction of nanoparticles and RBCs was studied (Avsievich et al. 2019, 2023). The group of Fedyanin studied RBC aggregation at System Lupus Erythematosis and highlighted four different RBC disaggregation scenarios, which could be used to characterize RBC properties (Khokhlova et al. 2010a, b, 2012). The group of Priezzhev measured RBC aggregation forces (Maklygin et al. 2012; Semenov et al. 2022), demonstrated its dependence on cell overlapping area (Lee et al. 2016b), compared RBC aggregation in autologous plasma and serum establishing the role of fibrinogen in this process (Lee et al. 2016a, c; Semenov et al. 2020) as well as the role of gamma globulins and its synergetic effect with albumin (Lee et al. 2017a). It was shown that RBC aggregation increases linearly with an increase in fibrinogen concentration (Ermolinskiy et al. 2024b). Aggregation of young and old RBCs was compared and RBC aggregation forces were measured at flow conditions (Lee et al. 2017b), RBC aggregation in dextran polymer solutions was characterized (Semenov et al. 2019). RBC aggregation was shown to decrease in the presence of endothelial cells pretreated with L-Arginine due to its nitric oxide production (Maksimov et al. 2024). The group of Wagner studied RBCs in flow, which allowed rapid change of suspending media for trapped cells (Steffen et al. 2011). This technique was applied to study the impact of calcium on RBC-RBC interaction in the context of clot formation. RBC aggregation was also studied by two oscillating traps, bringing the cells in contact (Kaestner et al. 2012). Novel research combines numerical simulations with optical tweezers experiments to investigate RBC aggregation and disaggregation. The study introduces a mobile-bridging model and shows that while aggregation is mainly governed by depletion interactions, RBC separation is best explained by a combination of depletion and mobile bridging mechanisms (Kaestner et al. 2012).

Optical tweezers are also used to evaluate nanoparticle-induced changes in RBC aggregation by measuring aggregation and disaggregation forces and the characteristic aggregation time, demonstrating that nanoparticles significantly alter these microrheological parameters (Korneev et al. 2026).

### Adhesion forces measurements

RBC adhesion to endothelium occurs in capillaries and impacts blood microrheology. Its alteration at such pathologies as sickle cell disease impairs oxygen transport and may even cause vasoocclusive events. First applied to characterize bacterial and fungal cell-surface interaction, optical tweezers were widely used for adhesion forces measurements in microrheological studies as well. RBC adhesion forces to surfaces were studied, for example, forces of adhesion to chitosan yarns were measured and ranged from 3.82 to 3.93 pN (Chen et al. 2018).

RBC-endothelium interaction includes both the exchange of signaling molecules and RBC adhesion to the endothelium. The latter is commonly considered non-significant at physiologic conditions, however at such pathologies as sickle-cell disease and diabetes mellitus, it is increased and can cause negative consequences (Wautier and Wautier 2013, 2020). Previously utilizing optical tweezers, we demonstrated non-zero RBC-endotheliocyte adhesion forces ranging from 1 to 10 pN (Kapkov et al. 2021). These forces were shown to depend on fibrinogen concentration and are considered of similar nature to that of RBC aggregation, namely depletion and bridging forces (Hebbel 2008; Kaliyaperumal et al. 2019). Later, the RBC adhesion to the cells of endothelium monolayer was studied (Ermolinskiy et al. 2024b). The forces ranged from 0.5 to 5 pN and also depended on fibrinogen concentration, however, the dependence was not linear and the forces reached plateau of approximately 3 pN after exceeding the physiological fibrinogen concentration (> 4 mg/ml). On the opposite to RBC aggregation, RBC-endothelium adhesion does not significantly depend on nitric oxide concentration (Maksimov et al. 2024). Recently, the forces of RBC adhesion to antigen-coated surface were measured (Lin et al. 2022). This study suggests the estimation of RBC surface antigens variation by optical tweezers**.**

### Other

Optical tweezers allow for combining with such techniques as Raman spectroscopy and fluorescent microscopy to obtain single-cell spectra (Xie et al. 2002; Avsievich et al. 2020; Barkur et al. 2020). It was applied to study malaria-infected RBCs and the impairment of oxygen transport by these cells was demonstrated (Dasgupta et al. 2011). Also, single-cell scattering properties can be measured to evaluate cell condition, sub-cellular components, etc. (Watson et al. 2004). The advantage of this technique compared to flow cytometry method is the ability of long-term single-cell measurements, which can be implemented for the external stimuli alterations (such as introduction of chemical compounds, etc.). Furthermore, Ramser et al. combined optical tweezers, Raman spectroscopy and microfluidics system to study single cell response to different conditions at constant flow (Ramser et al. 2005). Optical tweezers and microfluidics were also combined by Yao et al. (Yao et al. 2020). Optical traps can be coupled with UV-lasers to cut cellular organelles. For example, opto-injection technique allows precise drug delivery inside the cell membrane after its penetration by UV-laser beam (Zhang and Liu 2008; Stilgoe et al. 2024). Optical tweezers can be used to evaluate intracellular properties (Vos et al. 2024).

Optical traps are also widely used for cell transportation and cell sorting in the microfluidics systems (Zhang and Liu 2008; Avsievich et al. 2020; Yang et al. 2025). The technique of three-dimensional tissue printing involving optical trapping and transportation of cells advances rapidly (Mirsaidov et al. 2008; Natta et al. 2025). Optical tweezers are widely used to trap and manipulate cells in vivo at flow conditions (Zhong et al. 2013; Yu et al. 2020; Avsievich et al. 2020). Most of these studies are carried out on zebrafish or mice, since many vessels and capillaries there are available for visible and infrared light. It was possible to trap single cells and RBC aggregates inside the vessels, cause vasoocclusion by optical trap, etc.

Thus, optical tweezers provide vast opportunities for RBC studies. The main advantages of this technique are the absence of mechanical contact and the simplicity of sample preparation at most cases. It allows simultaneous trapping and dragging of several to dozens of cells with the optical forces ranging from 0.1 to 100 pN. The limitations of this technique are relatively slow processing time, high skill required for tweezers operating and the force limit of a hundred pN.

## Laser aggregometry of RBC and platelets

The blood studies began long before the invention of laser. First methods included microscopy and lamp light (Baskurt et al. 2011). In the seventies, the first works began to appear, where a laser beam scattered by platelets was studied (Broersma et al. 1973).

The method of laser aggregometry is based on the analysis of changes in the intensity of light scattered by a blood sample. During platelet or RBC aggregation, the average size of scattering particles increases, which causes an increase in the intensity of light scattered forward and a decrease in the intensity of light scattered backward (Yaroslavsky et al. 2002). Thus, changes in the intensity of scattered light can be used to determine the rate of aggregation and other dynamic parameters. It is important to note that the laser radiation does not significantly affect the cells. The wavelength of the laser used is selected to match the transparency window of the blood cells, so that the cells do not absorb the amount of light sufficient to cause significant heating or phototoxicity (Blázquez-Castro 2019). For RBCs, these wavelengths are in the range of 790–1064 nm (Krasnikov et al. 2011), for platelets: 550–900 nm (Meinke et al. 2007).

Recently, there has been a significant development in the field of laser aggregometry for RBCs. Commercially available instruments have emerged allowing the measurement of red blood cell aggregation both in flow and at rest. Furthermore, we describe some of these instruments: LORCA (Laser-assisted Optical Rotational Cell Analyzer, RR Mechatronics, Hoorn, The Netherlands), RheoScan (RheoMeditech, Seoul, Korea), Rheodyn SSD (Myrenne GmbH, Roetgen, Germany).

**Fig. 2:**
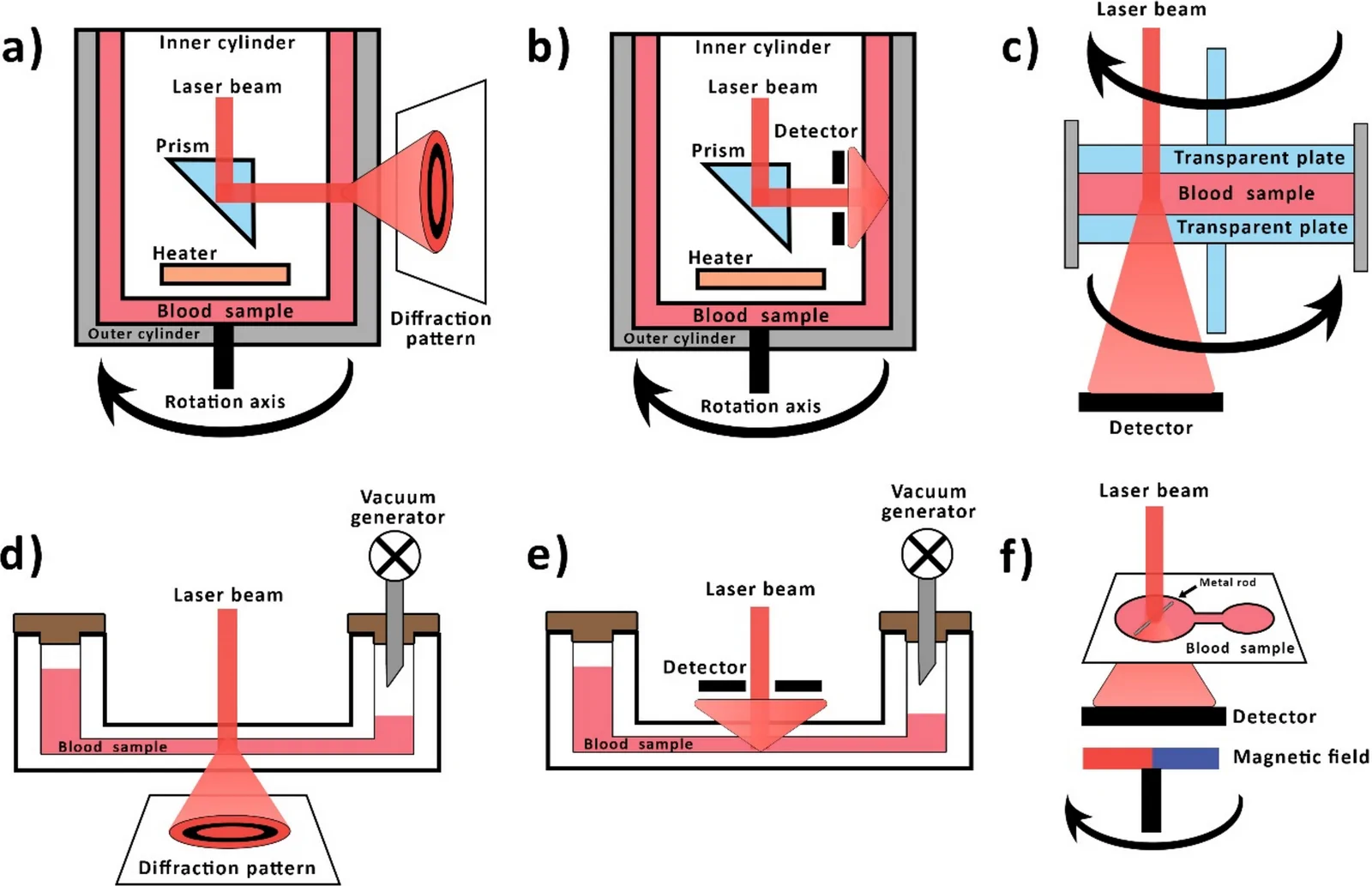
Schematic illustration on working principles of different ektacytometers and laser aggregometers. **a** LORCA ektacytometer, blood sample is placed in the gap between two coaxial cylinders, rotation creates shear stress and the diffraction pattern is analysed. **b** LORCA aggregometer, same scheme as (a) but backscattered light is analysed. **c** Myrenne ektacytometer/laser aggregometer, the blood sample is placed between two parallel transparent plates, rotation of which creates shear stress. Moreover, both plates can rotate at the same speed but in opposite directions, which creates unique measurement conditions. Either diffraction pattern or forwardscattered light is analysed. **d** RheoScan ektacytometer, transparent plastic cuvette with rectangular channel (~ 200 um) is irradiated by laser beam and the diffraction pattern is analysed. Shear stress is created via vacuum generator pushing blood suspension through the channel at varying speed. **e** RheoScan laser aggregometer, which measures RBC aggregation at flow conditions. Same as (d) but the backscattered light intensity is analysed. **f** RheoScan laser aggregometer, which measures RBC aggregation at static conditions. Laser beam irradiates transparent plastic cuvette with round channel filled with blood. The metal rod is placed into the channel by manufacturer, which can be spinned by alternating magnetic field created in the device. The rod rotation creates shear stress dispersing the RBC aggregates. When the rotation is ceased the RBC aggregation kinetics at stasis conditions is registered via measuring the forwardscattered signal. Adopted from (Schmid-Schönbein et al. 1996; Hardeman et al. 2001; Shin et al. 2005b)

The LORCA device was introduced by Hardeman et al. in 1994 (Hardeman et al. 1994). The RBC aggregation measurement system uses a Couette setup — made up of two concentric cylinders with a 0.3 mm gap — which can hold approximately 1 mL of whole blood (Fig. 2b). A stepping motor controls the rotation of the outer cylinder at adjustable speeds. The stationary inner cylinder houses a laser diode (670 nm, 4 mW) as the light source, along with a photodiode sensor and a temperature control unit.

**Fig. 3:**
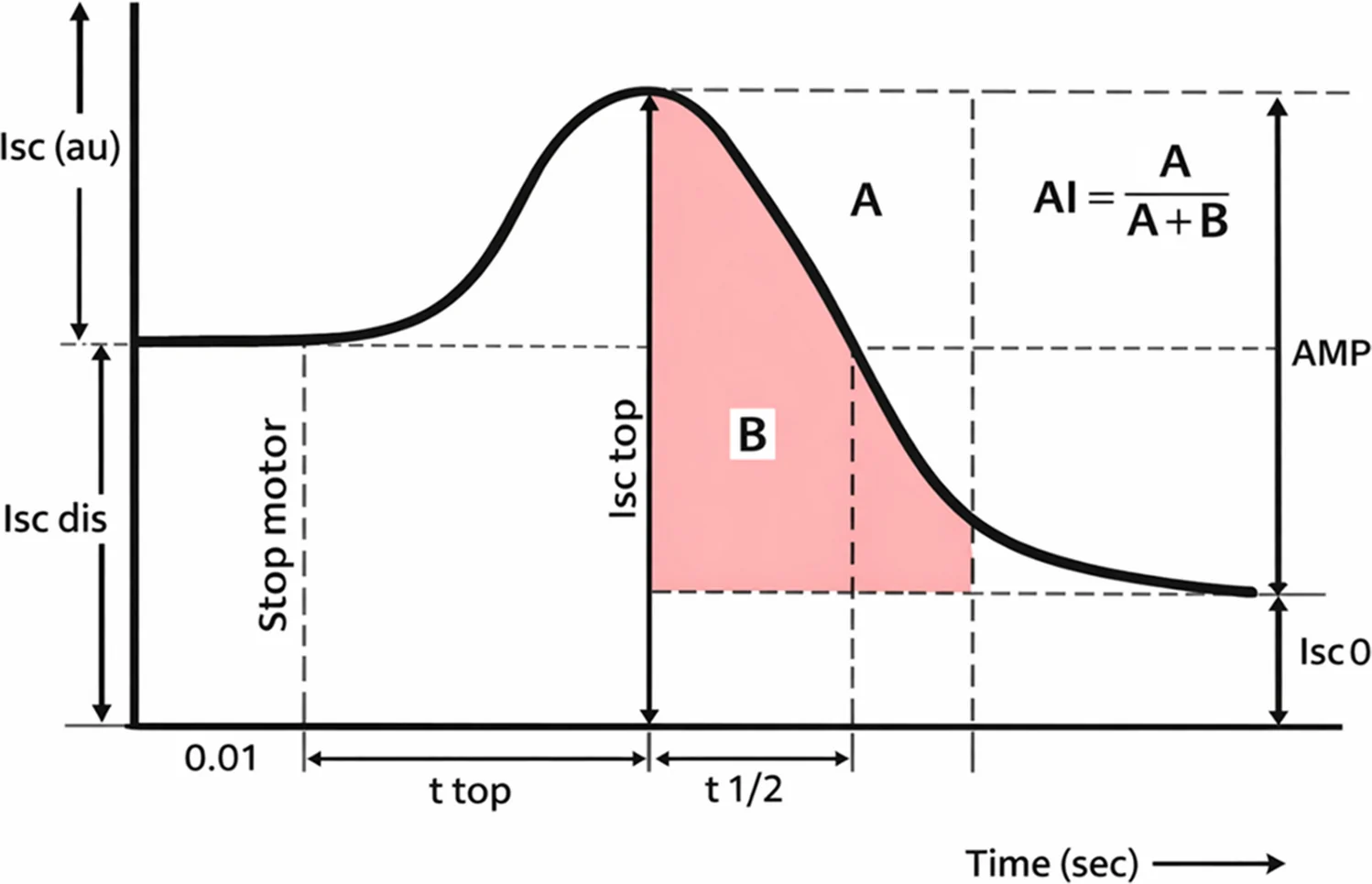
Typical syllectogram of RBC aggregation in LORCA device on logarithmic scale. Isc – intensity of backscattered light. Adopted from (Hardeman et al. 1994)

Figure 3 shows a typical syllectogram (the dependence of the intensity of backscattered light on time) obtained in the experiment. At the beginning of the measurement, the motor starts rotating the outer cylinder. The resulting shear stress destroys the aggregates that have formed in the gap after the administration of whole blood. After a few seconds, the cylinder stops abruptly, and the light backscattering increases due to a change in the orientation of the red blood cells (t < t_top_). Then the process of spontaneous aggregation of RBC in stasis begins and, as a result, the intensity of the light scattered back decreases. Subsequent analysis of the intensity drop curve allows us to obtain the following parameters:

1. AMP (in au) – amplitude, total extent of aggregation;
2. T_1/2_ (in s) – the time required for half of the RBCs to aggregate;
3. AI (in %) – the percentage of cells aggregated during the first 10 s:

The device is actively used by various scientific groups to study changes in blood aggregation parameters in patients suffering from various diseases. For example, the authors of Kuznetsova et al. (2022) identified increases in AI and AMP parameters in patients with polycythemia vera and stroke. Another scientific group investigated the effect of zinc on red blood cell aggregation in beta–thalassemia (Sinan et al. 2023). Zinc supplementation was shown to reduce red blood cell aggregation.

**Fig. 4:**
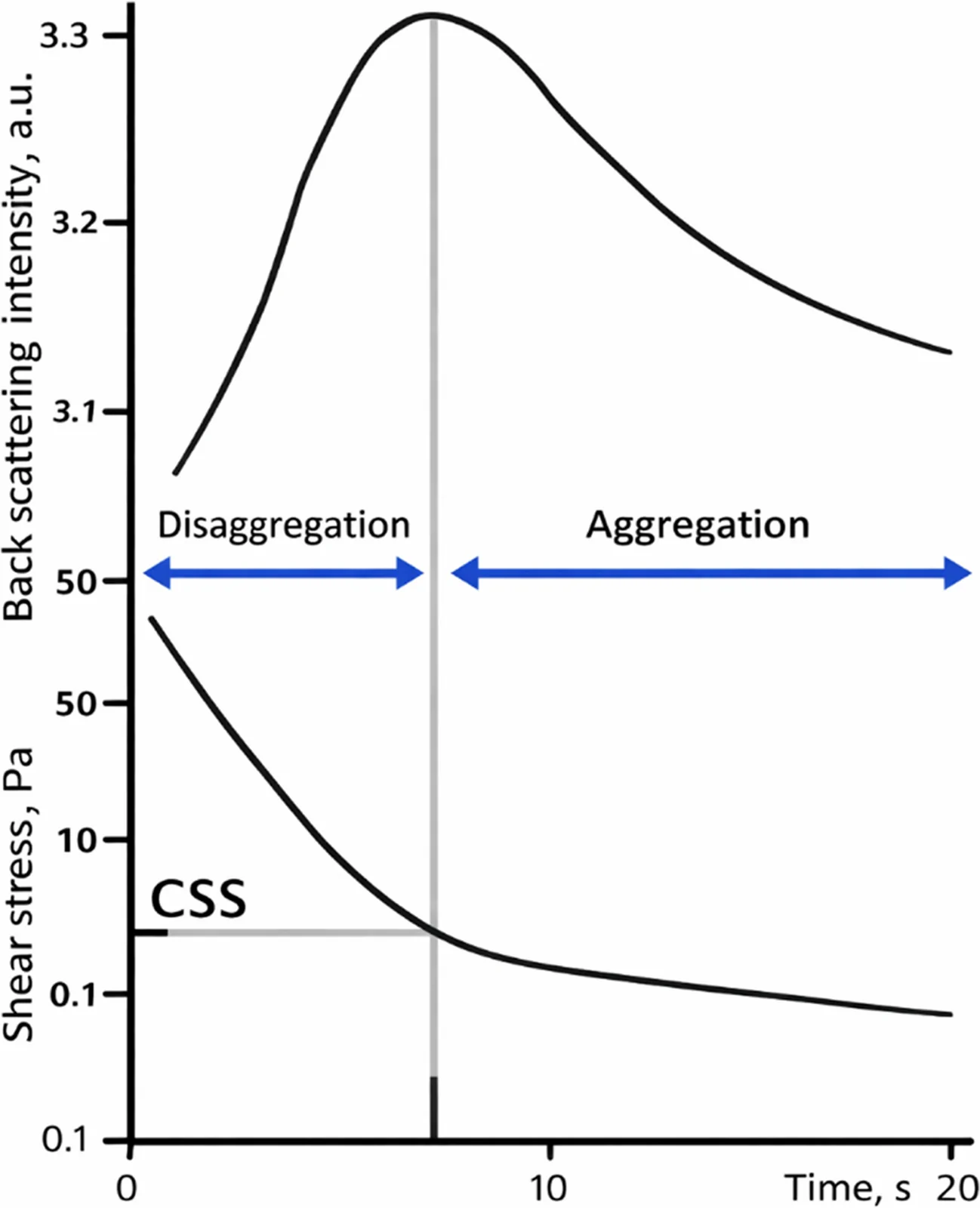
The time dependencies of backscattered light intensity (upper graph) and shear stress inside the channel (lower graph) with indicated areas. Adopted from (Ermolinskiy et al. 2023)

The RheoScan device developed by Shin et al. enables the study of RBC aggregation under both flow and static conditions (Shin et al. 2009a, b). The flow-based aggregation measurement system comprises a disposable transparent plastic element, a laser diode (λ = 635 nm, 1.5 mW), a photodiode, a pressure sensor, and a vacuum-generating mechanism (Fig. 2e). The disposable component features a microchannel (0.2 mm height × 4.0 mm width × 40 mm length) flanked by reservoirs at each end. For measurements, 500 μL of whole blood is introduced into the cuvette. A pressure differential is then established between the reservoirs via vacuum generation, inducing blood flow through the microchannel. A laser irradiates the central region of the microchannel, while the device simultaneously records pressure variations and backscattered light intensity by the flowing blood. A representative selectogram obtained from these measurements is presented in Fig. 4. The resulting curve exhibits two distinct phases. The initial intensity increase reflects a high-shear regime where shear stresses within the microchannel are sufficient to disrupt RBC aggregates. Subsequent intensity decrease occurs as the pressure differential declines, reducing shear stresses below the threshold required to maintain disaggregation. Consequently, RBCs begin to aggregate, leading to a decline in scattered light intensity. The extremum (peak) in the intensity profile corresponds to the equilibrium point between RBC aggregation and disaggregation processes. Evaluating the value of the critical time, one can assess critical shear stress (CSS) — shear stress at which the processes of RBC aggregation and disaggregation sustain dynamic equilibrium, thus the number of RBC aggregates formed in the unit of time equals the number of dispersed RBC aggregates. (Semenov et al. 2022). CSS characterizes the hydrodynamic strength of RBC in the blood suspension, because CSS can be considered as aggregating force between RBC (Shin et al. 2007a). Another obtained parameter is T_crit_ — time point of the CSS. It is important to mention that the graph of shear stress shown (Fig. 4) is theoretically calculated using measured pressure and determined cuvette parameters (Hou and Shin 2008).

Another part of RheoScan device also allows to measure aggregation of RBCs at static conditions (Shin et al. 2009b). This part is equipped with magnetic rotational mechanism, diode laser and a photodiode, which measures the transmitted light intensity (Fig. 2f). Measurements are performed as follows. Whole blood (8 μl) is placed in a thin (500 μm) microcuvette with metal bar inside, which can be rotated by a magnetic rotor. Right after the beginning of the measurement, the stirring bar starts to rotate at high speed, dispersing all aggregates formed at stasis conditions inside the cuvette. During this process the registered scattered light intensity of laser decreases (t < 0). At t = 0, the bar rotation stops. Then, the process of spontaneous aggregation of RBCs at stasis (without applied shear stress) in the blood sample occurs. Forward scattered light intensity monotonically increases, as the size of the RBC aggregates monotonically increases.

**Fig. 5:**
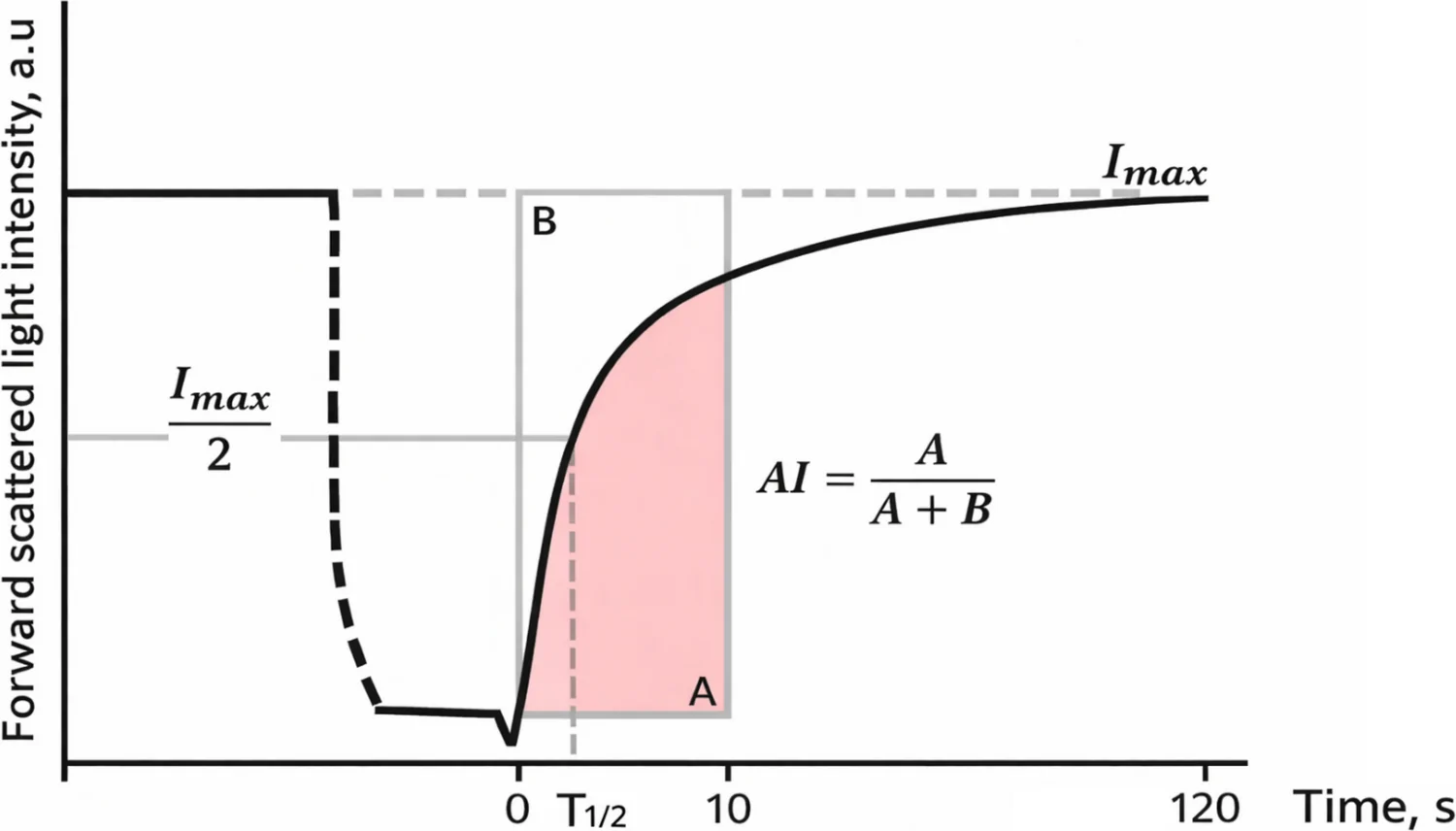
Aggregation kinetics and aggregation indices (Lugovtsov et al. 2019)

The device calculates the following parameters: T_1/2_, which characterizes the time of aggregate formation calculated as the time during which the intensity of light scattered forward reaches half of the saturation intensity value at maximum aggregation after 120 s (Fig. 5); parameter AI (aggregation index) characterizes the percentage of RBCs aggregated during the first 10 s. It is calculated as the ratio between the area under the aggregation curve and the whole area below and above the curve during the first 10 s of spontaneous aggregation process (Area B, Fig. 5); Amplitude (AMP): The difference between the I_min_ and the light intensity at either 10 or 120 s, indicating the extent of RBC aggregation (AMP_10_, AMP_120_). M-index: The area below the aggregation kinetics over 10 s time period (Fig. 5, area A), indicating the degree of the accumulated aggregation for 10 s (M).

Using the Rheoscan device, many results on RBC aggregation were also obtained. In particular, previously we have shown that in patients with type 2 diabetes mellitus, the hydrodynamic strength of aggregates and the rate of aggregation increase (CSS and AI parameters, respectively) (Umerenkov et al. 2024). Another group has studied the effect of remote ischemic conditioning on the aggregation RBC in patients with acute ischemic stroke (Blauenfeldt et al. 2025).

Another commercially available aggregometer is Rheodyn SSD (Myrenne GmbH, Roetgen, Germany) (Kiesewetter et al. 1982). The technology was initially developed by Bessis and Mohandas (Bessis 1975) and later adapted into a commercial device by Myrenne GmbH. The device is composed of a transparent upper plate and a transparent rotating lower cone, which together form the shearing unit (Fig. 2c). An infrared diode is positioned above the plate, while an infrared detector is placed beneath the cone (Kiesewetter et al. 1982). A suspension of red blood cells is introduced into the gap between the cone and plate, where it undergoes initial shearing at 500 s⁻^1^ for 10 s to break up any pre-existing aggregates. The shear rate is then stopped or reduced to a low level (3 s⁻^1^), and the forward-scattered light is measured. The instrument operates in a “10-s mode,” where forward-scattered light is recorded for 10 s, and the built-in microcomputer integrates the light transmission over time to determine the area under the curve. The results are reported as M (aggregation at stasis) and M1 (aggregation at 3 s⁻^1^), where higher values of both parameters indicate increased aggregation.

A study by Baskurt et al. (2009) conducted a systematic comparison of the aforementioned devices. The results demonstrated that the devices generally produce similar results when measuring the same blood samples. However, subtle variations in red blood cell aggregation were detected across devices, suggesting differences in sensitivity or methodological approaches. Notably, challenges arose when assessing samples exhibiting either markedly elevated RBC aggregation or impaired cellular deformability.

An alternative method for assessing red blood cell aggregation is based on the erythrocyte sedimentation rate (ESR). However, since the ESR is influenced by multiple factors, this approach only permits an indirect evaluation of erythrocyte aggregation (Priezzhev et al. 2016). Nevertheless, this technique remains widely utilized in clinical settings as part of standard hematological testing (Tishkowski and Gupta 2025).

Ultrasound-based methods are also widely used to study erythrocyte aggregation. These methods are based on the analysis of the intensity of an ultrasonic wave backscattered from a blood sample in vitro or in vivo (Yoon 2017; Bok et al. 2025). This approach provides information on the average aggregate size, compactness, aggregation degree, and other parameters (Rouffiac et al. 2002; Rouyer et al. 2018; de Monchy et al. 2018).

Laser-based techniques for assessing platelet aggregation hold significant diagnostic potential and continue to evolve. Among these, turbidimetric and fluctuation-based methods are the most widely employed. The turbidimetric approach, originally developed by Born and O'Brien in 1962, remains the gold standard for evaluating both induced and spontaneous platelet aggregation by measuring changes in platelet rich plasma optical density (O’Brien 1962). This method relies on the principle that platelet aggregate formation reduces the total light-scattering surface area of cells, thereby altering the optical properties of the sample (Born 1962).

**Fig. 6:**
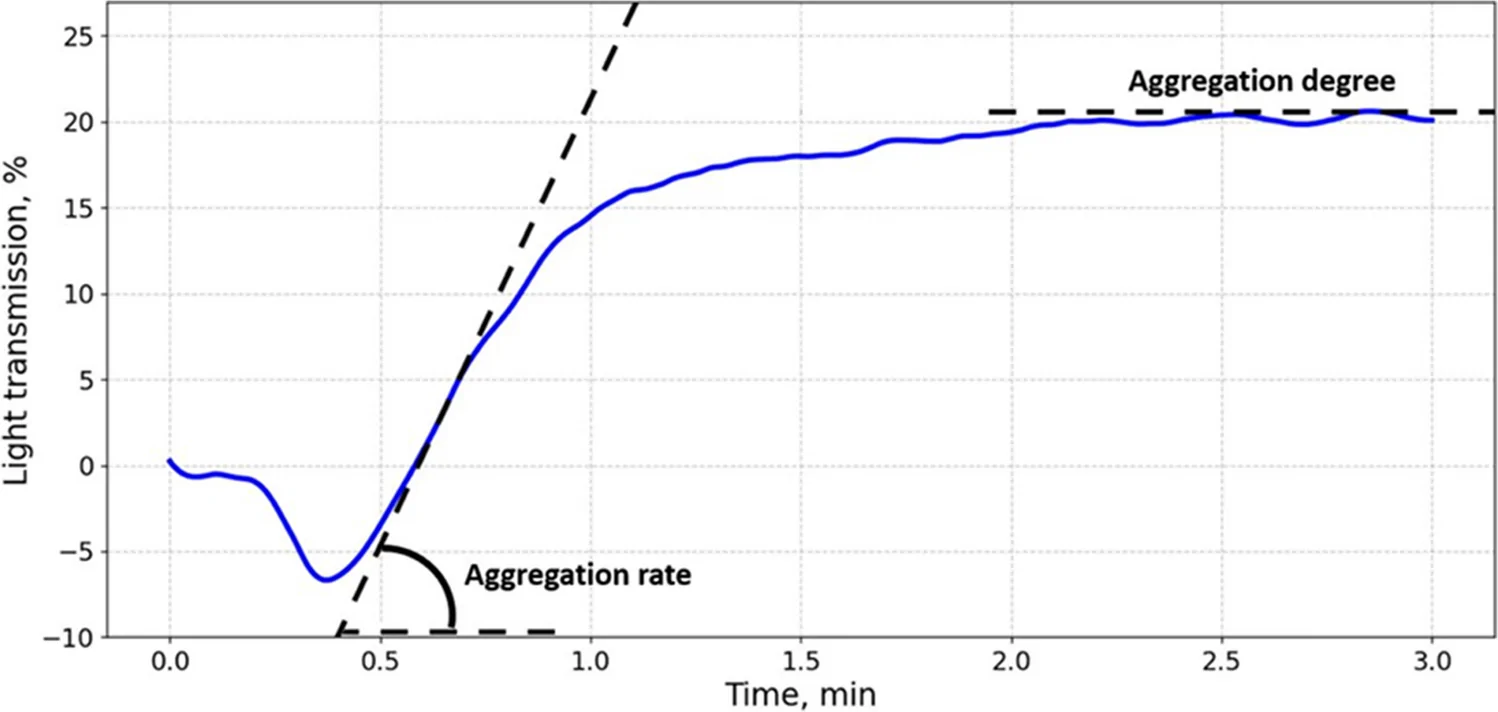
Typical aggregogram obtained during the experiment with platelet rich plasma

While turbidimetry enables the assessment of platelet aggregation dynamics and morphological changes (e.g., shape transformation), it has notable limitations. For instance, shape change artifacts may obscure the initial phases of aggregation (Maurer-Spurej and Devine 2001). Furthermore, the conventional Born method lacks sensitivity in detecting small aggregates (comprising fewer than 50–100 platelets), potentially leading to underestimation of early aggregation events (Paniccia et al. 2015). Key Parameters in Aggregogram Analysis: the degree of aggregation (maximum aggregation achieved (%)), the rate of aggregation (the angle of the curve (Fig. 6) at the stage of rapid aggregation).

The fluctuation method was proposed in 1989 by Z.A. Gabbasov and co-authors (Gabbasov et al. 1989). It is based on the analysis of fluctuations in light transmission caused by a random change in the number of particles in the optical channel. The relative dispersion of such fluctuations is proportional to the average size of aggregates and is used to study the kinetics of aggregation. The main parameter of the method is calculated using the formula:

1 $${\left(\frac{\upsigma }{I}\right)}^{2}=\uptheta \hspace{0.17em}\frac{{\sum}_{n}{K}_{n}^{2}\hspace{0.17em}{N}_{n}\hspace{0.17em}n\hspace{0.17em}{R}_{n}}{{\sum}_{n}{N}_{n}\hspace{0.17em}n}$$

where σ – standard deviation of light transmission, I – average light transmission value, θ – total platelet volume, K – scattering efficiency, N – number of platelets, R – the radius of an aggregate of n platelets (Filkova et al. 2019).

The method is highly sensitive, which makes it suitable for the study of spontaneous aggregation, aggregation under the influence of low concentrations of inducers, as well as aggregation of subcellular particles and macromolecules. The particles are classified by size as small (9–25 µm), medium (25–50 µm) and large (50–70 µm). However, this method has a number of disadvantages. The method cannot distinguish whether different statistical behaviors occur in different portions of the time traces, limiting dynamic interpretation. Also, it requires high signal-to-noise ratio, as unstructured measurement noise can obscure intrinsic fluctuations (Coulon and Larson 2016). Special signal processing software is also required to implement this method.

There are several commercially available instruments used for the study of platelet aggregation, such as the BIOLA ALAT-2 (Biola Ltd., Moscow, Russia), SOLAR AP2110 (SOLAR Laser Systems, Belarus) and the Chrono-Log Model 700 (Chrono-Log Corporation, USA). These devices differ in their methodological approaches to platelet aggregation analysis, sample requirements (e.g., platelet rich plasma or whole blood), the number of measurement channels, and other technical specifications.

The structural and functional design of the device will be examined using the BIOLA ALAT-2 (Biola Ltd., Moscow, Russia) (Gabbasov et al. 1989) as a prominent example. This instrument employs both turbidimetric and fluctuation-based analytical methods to assess platelet function. The device features a semiconductor laser with an output power of 1–50 mW and a wavelength of 785 nm, a thermostatically controlled measurement chamber housing a glass cuvette, and a photodetector to record changes in light transmission through the sample. For measurements, 300 µL of platelet rich plasma (PRP) is introduced into the cuvette and incubated at 37 °C for 3 min to ensure thermal equilibration. Prior to measurement initiation, a freely rotating metal stirrer is placed inside the cuvette to maintain uniform platelet suspension. Upon starting the assay, the stirrer rotates at 800 rpm, ensuring consistent mixing of the sample. To induce aggregation, 15 µL of 5 μM adenosine diphosphate (ADP) is added 10 s after measurement commencement. The resulting changes in light transmission are recorded in real time, producing a characteristic aggregation curve (aggregogram), as illustrated in Fig. 6.

The initial state is assumed to be 0% aggregation — the first 10 s after the start of the measurement before the addition of ADP to PRP, and the condition of platelet poor plasma (PPP) is assumed to be 100%. At the same time, with sufficient accuracy for practice, it can be assumed that the optical properties of PPP and distilled water are equivalent (on the ALAT-2 device, 100% calibration is performed with distilled water). The greater the intensity of the light scattered forward, the larger the scattering particle relative to the wavelength of the light. A “dip” of 10 s corresponds to the addition of ADP (Gabbasov et al. 1989). Thus, the degree of aggregation (maximum light transmission signal for 5 min) and the rate of aggregation (maximum slope of the light transmission curve for 5 min) are calculated.

**Fig. 7:**
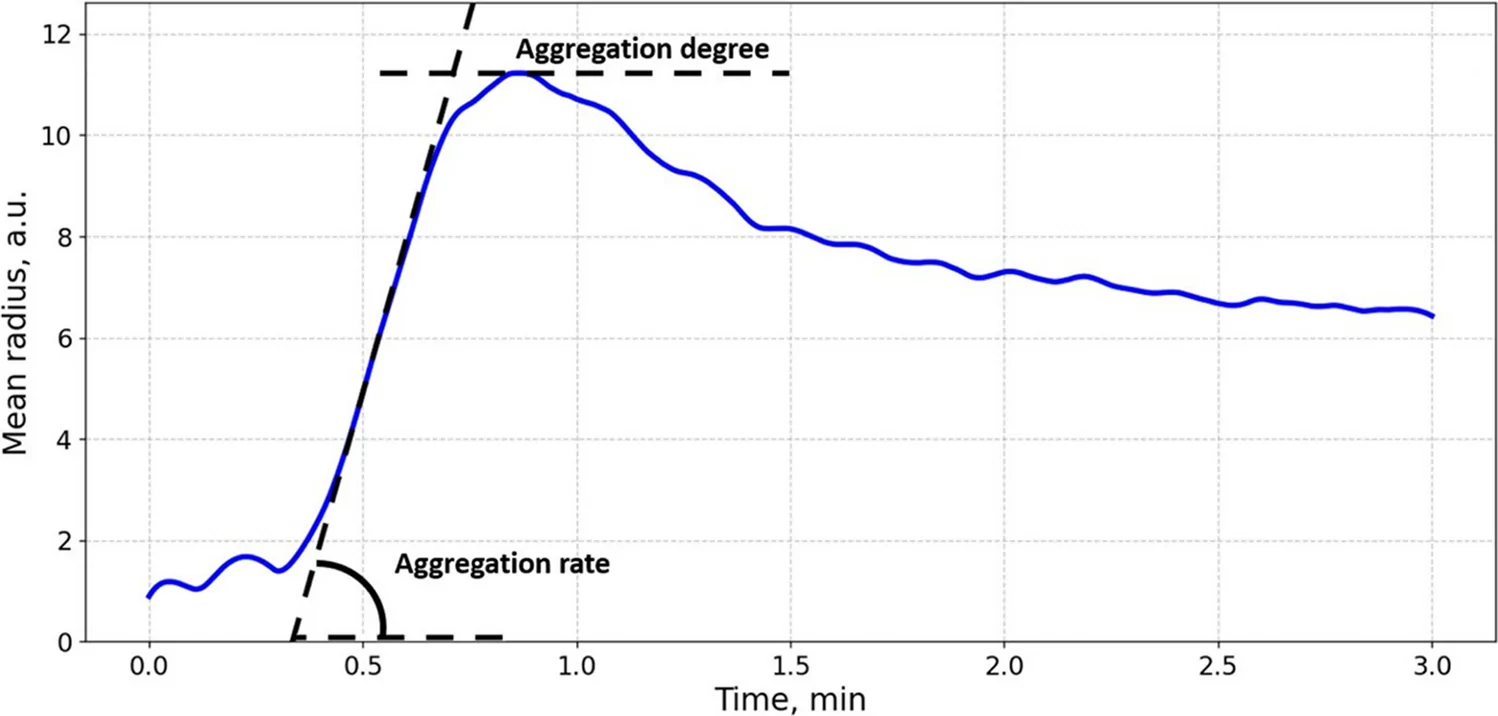
Typical dependence of the calculated mean radius of the particles on time, obtained in the BIOLA measurement

Platelet aggregation parameters can be quantitatively assessed by measuring the mean radius of light-scattering particles. This radius is derived from fluctuations of the intensity transmitted through the platelet suspension, as described by formula 1 (Filkova et al. 2019). During the aggregation process, the mean radius of the particles increases due to the formation of platelet aggregates, which significantly exceed the size of individual cells. By analyzing the curve (Fig. 7) key parameters can be obtained: aggregation rate (a.u.) — determined by the maximum slope of the curve; degree of aggregation (a.u.) — defined by the maximum radius of scattering particles.

Using this device, differences in platelet reactivity in patients with acute ischemic stroke treated with aspirin were studied (Ikonnikova et al. 2022). Some patients were found to be resistant to aspirin. In another work the dynamics of coagulopathy in patients with different COVID-19 severity was studied (Kalinskaya et al. 2020). It was shown that patients with COVID-19 have platelet hyperactivation, especially in severe cases of the disease.

A comprehensive evaluation of the discussed devices reveals distinct technical and operational characteristics that influence their suitability for various applications. Among these systems, the Solar AP2110 exhibits the closest technical alignment with the BIOLA device, as both instruments utilize turbidimetric methodology for platelet aggregation assessment. This shared analytical approach requires PRP as the test medium, introducing additional preparatory steps that extend the overall processing time. In contrast, the Chrono-Log Model 700 employs an advanced dual-method system combining optical density and impedance measurements, enabling direct analysis of whole blood samples without PRP preparation. While this instrument has been widely adopted in research environments and extensively documented in scientific literature, it presents certain limitations that must be acknowledged. The system demonstrates sensitivity to methodological variations, including differences in agonist concentrations, specimen handling protocols, and interpretation criteria—a challenge common to optical aggregometer platforms (Le Blanc et al. 2020). Furthermore, despite its dual-channel configuration, the device’s throughput capacity remains insufficient for high-volume clinical laboratory operations. For clinical applications requiring standardized, high-efficiency analysis, automated systems such as the Sysmex CS-2000/CS-2500 series (Norderstedt, Germany) have proven to be particularly effective and are consequently preferred in large-scale clinical trial settings (Le Blanc et al. 2020).

## Optical microscopy enhanced with flow chamber and artificial intelligence

Earliest microscopic studies of RBCs allowed morphological assays but lacked the flow natural to RBCs travelling in vessels. Blood flow creates shear stresses applied to the cells, which cause their deformation and alter cell properties. To overcome this limitation the flow chamber method was introduced. Basically, the microscope is coupled with a cuvette, in which the flow is generated. This allows for observing the deformed cells while varying the values of shear stresses applied. This method not only allows morphological studies at in vitro system approaching in vivo conditions but also enables estimations of RBC deformability. From the moment of first applications of this technique in 1970 s up to date the technology has advanced vastly. For example, complex microfluidic systems mimicking the capillary network were created and machine learning algorithms were introduced to simplify the measurements and conduct faster experiments.

The earliest publication on flow chamber-coupled microscopy applied to RBC studies, which we managed to track is that by (1973). The basic principles remained practically unchanged since then. To create a flow chamber one needs a channel, usually rectangular in shape, isolated from the environment. More complex flow chamber geometries can benefit the study (An et al. 2023). Chambers can be fabricated from glass or polymeric materials (e.g., polydimethylsiloxane—PDMS) using photolithography or 3D printing techniques (Shrirao et al. 2018). Two holes are created, inlet and outlet, the cell suspension is pushed through the inlet, the flow through the channel is observed with the microscope and leaves the chamber through the outlet. To achieve this, usually, a stepper motor coupled with syringe is used. The shear stresses applied to the cells depend on the channel geometry, suspension viscosity and flow rate. Basically, shear stress τ equals the suspension viscosity μ times flow rate gradient ∇υ.

The method is applicable both for studying deformability (Dobbe et al. 2002a; Londero and Riquelme 2021) and for investigating RBC aggregation (Tikhomirova et al. 2017; Muravyov et al. 2017; Mehri et al. 2018; Alexandrova-Watanabe et al. 2025), including the kinetics and morphology of aggregates. It allows for the study of the dynamic behavior and heterogeneity of large cell populations under controlled shear conditions, which is particularly valuable for understanding the mechanisms of pathological processes. Flow chambers are also used for studying the endothelium under flow (Fallon et al. 2021). Two possible approaches on RBC observation in flow chamber exist. First is the observation of cells moving in the flow, the second is observation of cells attached to the channel bottom deformed by the flow. While the first approach grants more physiologic conditions it also requires a complex high-speed camera capable of distinguishing the moving cells’ borders. On the other hand, the second approach represents cell adhesion, which does not occur naturally, however, the camera used can be much simpler (Artmann 1995). Another advantage of the latter approach is that the elongation of the same cell can be estimated at various shear rates. The alternative to the mentioned techniques is a microscope-coupled camera, focusing on a particular cell and following it at the same rate as the flow (Atwell et al. 2022). This allows to avoid the cell attachment to the surface and does not require a high-speed camera. When working with RBCs resuspended in a solution different than autologous plasma, it is necessary to consider the ‘glass effect’, which arises from the unnatural interaction of the cells with the flow chamber material. This effect, which impacts the cell adhesion to the surface, can be partially compensated by adding albumin at a low concentration, however, the standardized protocols for its optimization are still lacking (Londero and Riquelme 2021).

The cells attached to the chamber bottom allow not only for measuring the elongation index (for definition see the optical tweezers section) at various shear rates but also for measuring the relaxation time (time required to restore the initial shape after the flow stop) (Artmann 1995; Antonova et al. 2023). Compared with ektacytometry and other methods providing only the mean elongation index and its standard deviation, the flow chamber method distinguishes the values for single cells, which allows for visualizing cell deformability distributions. In turn, this allows for characterizing of cell subpopulations with abnormal deformability. The impact of such subpopulations on the mean deformability of all cells may be negligible, however, in vivo this impact can be significant leading to vasooclusive events and impaired microcirculation. Thus, the flow chamber can provide more valuable information on RBC deformability compared with ektacytometry.

RBC deformability distributions were obtained for blood before and after its preparation for long-term storage in blood banks (Barshtein et al. 2020). The deformability of density-fractioned RBC subpopulation was studied using the flow chamber technique in (Barshtein et al. 2024). Correlation of density and impaired deformability as well as the heterogeneities of each density subpopulation were demonstrated. The study (Carciati 2017) obtained images of individual infected and healthy RBCs moving over endothelial cells, demonstrating different dynamic cell behaviors. The flow chamber technique was applied to study the RBC response to gasotransmitters, namely, nitric oxide, hydrogen sulfide and carbon monoxide (Muravyov et al. 2021). The complex system of interconnections between gasotransmitters was revealed and their effect on RBC deformability was demonstrated (Muravyov et al. 2019; Maksimov et al. 2024). Flow chamber was coupled with optical tweezers and fluorescent microscopy to study malaria-infected RBCs (Bambardekar et al. 2008). More complex flow configuration creating the same shear stress values across the channel was reported and used to study the iron deficiency anemia RBCs (Elblbesy 2018). Flow chambers also can be used to study RBC aggregation dynamics (Chen et al. 1994).

The use of optical microscopy in microfluidic systems provides direct real-time visual observation of cells, creating an ideal foundation for applying machine learning algorithms for image analysis. Numerous effective open-source neural network frameworks exist that can serve as a basis for developing custom software for cell counting and morphology analysis: Cellpose (Stringer et al. 2021) (for deformability), Omnipose (Cutler et al. 2022) (for aggregation), LimTracker (Aragaki et al. 2022) (for tracking), Svetlana (Cazorla et al. 2024) (for classification), iCLOTS (for clustering) (Fay et al. 2023), among others. Neural network approaches significantly enhance the speed and accuracy of image analysis compared to traditional methods (such as manual processing, threshold segmentation, and correlation tracking), especially on low-quality microscope images. It is particularly noteworthy that during training, neural networks identify features in images that are not always discernible by the human eye (Lamoureux et al. 2022; Link et al. 2023).

Methods similar to flow chamber include the following techniques. (1) Microfluidics, which allows the observation of cells travelling through complex microchannel systems mimicking the capillary network (Grigorev et al. 2023). Among the possible measured parameters are transit speed and time and frequency of stasis events, when the cells block the flow through one of the channels. (2) Filtration techniques, in which the cells squeeze through narrow pores and the transition time associated with cell deformability is measured. For example, see cell transit analyser method (Fisher et al. 1992). (3) Rheoscopy, including a device in which two parallel planes rotate at same rate in opposite directions (Dobbe et al. 2002a, b). Due to counter-rotation the cells do not change their position and deform by the shear stress created. This allows the usage of regular camera for cell observation, however, the precise rotation control system is required. (4) Quantitative phase microscopy, which allows nanometer-scale observations of RBC membrane fluctuations (Popescu et al. 2008; Kim et al. 2019). The data obtained can be used to estimate cell deformability. (5) Resistive pulse sensing also known as Coulter counter technique, a method based on the measuring electric signals from cells passing through the narrow pores (Don 2003; Igout et al. 2004; Taraconat et al. 2019). This allows for assessing cell number, size and elongation. For more detailed data on the techniques listed see references (Musielak 2009; Kim et al. 2015).

It is important to note the recent trend towards using microfluidic devices as flow chambers (An et al. 2023). Microfluidics offers several advantages: precise control over channel geometry and flows at the cellular level, chip miniaturization, small sample and reagent volumes, the ability to create complex configurations for specific tasks (e.g., for cell sorting), and the potential for integrating multiple functions on a single chip. Unlike traditional methods, microfluidic chips allow a precise control over the direction and velocity of the cell flow. Through the use of microfluidic channels and micropillar arrays, such systems can directionally influence the shape of individual cells (Piety et al. 2021; Kumari et al. 2024) or their aggregates (Deng et al. 2020). Microfluidic devices surpass traditional ektacytometers in sensitivity: as demonstrated in (Piety et al. 2021), the passage of RBCs through a network of microfluidic capillaries allows for more precise assessing the progressive decrease in their deformability during storage.

## Laser diffractometry

Beginning in the 1980 s, nowadays laser diffractometry (LD) has become a powerful method in the analysis of RBCs, providing insights into their size distribution, deformability, aggregation, and viscoelastic properties (Bayer et al. 1992). The technique's ability to measure parameters of RBC membranes at both stationary and dynamic conditions makes it a versatile tool in research and clinical applications (Evans et al. 1984). This method is especially valuable in assessing RBC deformability, a critical factor in blood fluidity and microcirculation, which is often impaired in various vascular pathologies, sickle cell disease and diabetes mellitus (Caimi and Lo Presti 2004; Riquelme et al. 2006; Shin et al. 2007b; Presti et al. 2012; Atwell et al. 2022). The refractive index of erythrocytes can also be measured using laser diffractometry, and this parameter was shown to differ significantly between healthy individuals and patients suffering from certain diseases, such as multiple myeloid diseases (Bessmeltsev et al. 2002).

The LD method is based on the effect of diffraction of a laser beam on a blood smear or a thin layer of RBC suspension. When a coherent light beam impinges on the boundary of an RBC a specific diffraction pattern, referred to as Airy pattern, is generated, which shape and size depend on RBC size, shape, and refractive index. The task of laser diffractometry is to determine the parameters of RBC ensembles based on measurements of the characteristics of the diffraction pattern. For more details on this, see (Nikitin et al. 2024). The advantage of the LD is that this method allows for rapid processing of large ensembles of RBCs without requiring complex equipment such as microscopes, microchannels, high-speed cameras, etc.

**Fig. 8:**
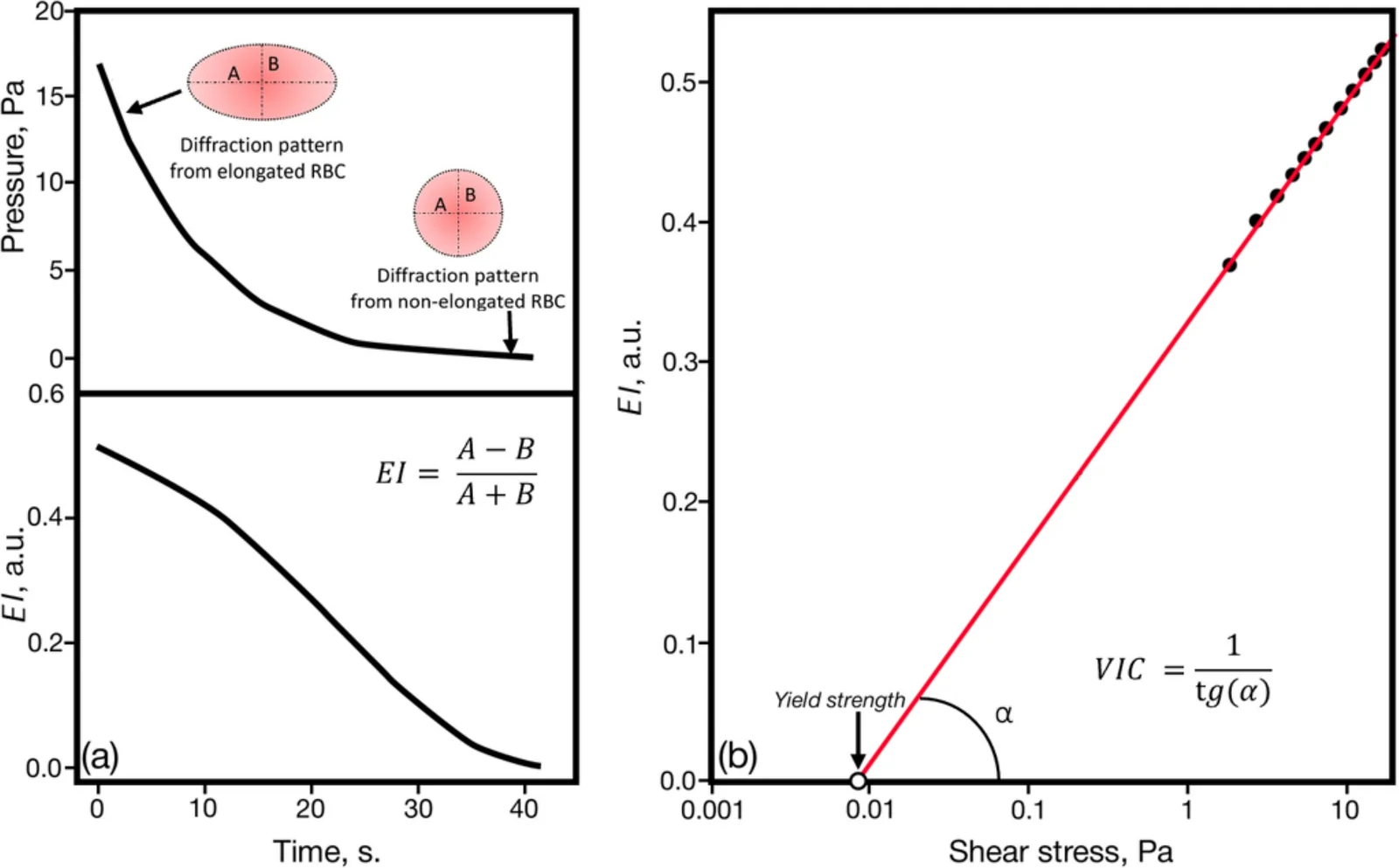
**a** (below) The elongation index (EI) as a function of time; (above) the pressure difference in the flow as a function of time. **b** EI as a function of shear stress in semi logarithmic axes. The explanation of Yield strength, i.e., the minimum shear stress required for RBC elongation, and RBC intracellular viscosity (VIC) calculation. α is the angle of slope of the curve. VIC is a dimensionless value characterizing the intracellular viscosity

One of the most notable technological advancements is the development of laser-diffraction ektacytometers based on LD. These devices usually incorporate a disposable element that holds the blood sample, making them easily usable in clinical settings. The design of these ektacytometers is simple, with no moving parts, and they offer low cost, short operating times, and ease of operation (Shin et al. 2005a; Parrow et al. 2018). This method analyzes the diffraction patterns obtained from a diluted RBC suspension illuminated by a laser beam. As the RBCs elongate due to shear stress arising under the pressure difference in the flow, the diffraction pattern from the cells also elongates, allowing for the calculation of the elongation index (EI) (Fig. 8a). This provides a quantitative measure of the cells’ ability to deform under different shear stresses. The relationship between EI and the shear stress on a semi-log scale can be approximated using linear regression, allowing the Yield strength and viscosity of RBC intracellular content (VIC) to be calculated (Fig. 8b). More detailed information about the determination of these parameters can be found in ref. (Firsov et al. 2006; Ermolinskiy et al. 2024a).

The analysis of data obtained via laser diffractometry was also refined. New mathematical models and algorithms have been proposed to process the diffraction patterns and extract relevant parameters, such as the elongation index (EI) and the shear stress required to achieve half of the maximum deformation (SS1/2) (Baskurt and Meiselman 2013). These models have improved the accuracy and reliability of the measurements, enabling better characterization of RBC deformability and aggregation. Ektacytometry has undergone significant advancements in recent years. New diffractometric equations and data processing algorithms have been developed to relate the parameters of the diffraction pattern to the characteristics of the RBC ensemble, such as mean deformability and the width and asymmetry of the deformability distribution (Nikitin et al. 2016, 2024). These advancements have improved the ability to characterize the deformability of RBCs in shear flow and to evaluate the statistical characteristics of inhomogeneous ensembles of cells.

While laser diffractometry offers significant advantages in RBC analysis, it is important to consider its limitations, such as the potential for measurement errors due to noisy diffraction patterns (Bayer et al. 1992; Nikitin et al. 2016). Additionally, the technique’s sensitivity to various factors like hematocrit levels and light intensity must be managed to ensure accurate results. Analysis of the obtained data can yield meaningless results if incorrect optical models are applied (Keck and Müller 2008). The technique provides only mean values of RBC size and deformability indices, which impairs its sensitivity to subpopulations of cells with altering properties. This can be partly overcome by applying algorithms, which provide such parameters as size dispersion or deformability dispersion (Nikitin et al. 2016, 2024). These parameters are calculated using certain features of the diffraction pattern. Samples with subpopulations of RBCs with anomalous properties demonstrate higher parameter dispersion and thus can be detected via laser diffractometry. However, the precise data on the subpopulation size and mean parameter values for each subpopulation are principally unavailable.

Laser diffractometry has proven to be indispensable technique in the analysis of RBCs, with applications ranging from basic research to clinical diagnostics. Its ability to provide detailed information on RBC size distribution, deformability, aggregation, and viscoelastic properties makes it an asset in hemorheology. As technology continues to advance, further improvements in hardware, data processing algorithms, and clinical applications are expected, ensuring that laser diffractometry remains at the forefront of RBC analysis.

Several commercial ektacytometers exist, such as the LORCA, Rheoscan, and Rheodyn SSD (described in the section “Laser aggregometry of RBC and platelets”), as well as the recently released MIZAR® (ALCOR Scientific, USA) and others (Groner et al. 1980; Bareford et al. 1985).

## Flow cytometry

Flow cytometry method is used for studying individual cells by measuring the light scattered on them when illuminated by a laser beam. Typically, a flow cytometer measures forward scatter (FSC) and side scatter (SSC) at a 90° angle to the incident light (Wagner and Flegel 1998; Dunne and Maecker 2019). The physical principle of flow cytometry is based on the dependence of laser light scattering on cell size, internal structure, and other properties (Wagner and Flegel 1998). FSC primarily correlates with the size of the cell, meaning that greater forward scatter corresponds to a larger cell size. SSC correlates with the granularity or internal complexity of the cell; in other words, a higher signal of side scatter indicates more internal structures (organelles, various inhomogeneities) within the cell. Typically, the particle stream is directed toward the center of the measurement capillary (with dimensions ranging from 70 to 150 µm) using hydrodynamic focusing. This process isolates individual cells from the other cells, causing them to pass through in single line. The laser wavelength is usually selected based on the specific experimental objectives [“Flow Cytometry: The Next Revolution”, “Flow Cytometry: Advances, Challenges and Trends”]. The intensity of FSC and SSC from the cells is measured in the experiment (Givan 2004). Additionally, fluorescence detectors are placed in the SSC channel to obtain supplementary and, in most experiments, essential information about the cells. Based on how the fluorescence signal is detected, two main types of flow cytometry are distinguished: polychromatic cytometry (PC), which assigns each fluorochrome to a specific detector, and spectral cytometry, which works with the whole spectra (Robinson et al. 2026). In the PC method, optical filters and dichroic mirrors are used to separate the light from different fluorophores into specific wavelength ranges. Each fluorophore is detected by its own detector, with signals being assigned to predefined detector channels. This method is limited by the number of usable fluorophores (approximately 30) due to spectral overlap and the finite number of available lasers. In spectral cytometry, the full emission spectrum of each fluorophore is analyzed using a grating or prism. Subsequently, computational unmixing algorithms are applied to mathematically separate the overlapping spectra. This approach enables the use of more than 50 fluorophores in a single experiment. Different types of tasks require different flow cytometer configurations, which vary in capillary size, lasers, detector placement, the detectors themselves, and other parameters (Givan 2004; Adan et al. 2017). All parameters are selected based on the cells studied. Sometimes, instead of a side scatter, a backscatter detector (BSC) is used in the flow cytometer’s optical setup, which detects light scattered in the backward direction (Kerker 1983). It is important to understand that the detected forward scatter correlates with cell size, but for certain size ranges, depending on the laser and detectors, it may be poorly sensitive to size variations (Givan 2004). A similar principle applies to side scatter. Therefore, measurements of the same cell type on one flow cytometer may yield more significant results than on another instrument with a fundamentally different optical configuration.

Flow cytometry allows for the measurement of fluorescent light emitted by a cell at different wavelengths. This capability enables the differentiation of various cell types or the detection of specific molecules and structures, such as fluorescently conjugated antibodies, fluorescent proteins, microparticles, and more (Dunne and Maecker 2019; Robinson et al. 2026). Consequently, this method finds wide application in immunophenotyping, where it is used to identify and characterize immune system cells, aiding the diagnosis of immunodeficiencies, monitoring immune responses, and classifying leukemias (Kwon et al. 2020; Li 2022; Robinson et al. 2026). The method is also extensively used for studying DNA, allowing the measurement of its content to determine ploidy (diploid/aneuploid cells) and cell cycle phase distribution (G1, S, G2/M) (Robinson et al. 2026). This can be applied in cancer research to identify DNA mutations. The flow cytometry method is often combined with other techniques: with microscopy (Imaging flow cytometry) to obtain an image of each measured cell (Gualda et al. 2017; Qiu et al. 2024); with mass spectrometry (Mass Cytometry (CyTOF)), where antibodies are labeled with heavy metals instead of fluorophores, and cells are analyzed using mass spectrometry (Bandura et al. 2009; Bendall et al. 2011; Bodenmiller et al. 2012); with FLIM methods (Fluorescence Lifetime Flow Cytometry) [High-throughput fluorescence lifetime imaging flow cytometry] and others (Robinson et al. 2023, 2026).

The method also has several limitations. Detecting viruses or bacteria can sometimes be challenging, as the size limit for measurable particles is approximately 200 nm (Müller and Nebe-von-Caron 2010). Sorting technologies based on flow cytometry also have a drawback: they can induce cellular stress, which may disrupt morphology or lead to internal changes (Robinson et al. 2026). Another significant issue is the lack of standardization and reproducibility of measurements between different instruments. This is primarily caused by different data processing software, the absence of a unified data format, and variations in the quantitative assessment of research results (Robinson et al. 2026).

Flow cytometry is a powerful tool for developing various functional assays that provide crucial information about cellular processes and behavior. It enables researchers to observe cellular processes and responses in real time, making flow cytometry an effective instrument for research in cell biology and immunology (Perfetto et al. 2004; Wlodkowic et al. 2011; Chattopadhyay and Roederer 2012). The method is also widely employed for sorting individual cells. Sorting is conducted based on fluorescence-based methods (Herzenberg et al. 2002), label-free approaches (Zhang et al. 2024), and microfluidic systems. Flow cytometers are widely used to study the properties of blood cells: RBC (Grisendi et al. 2015), platelets (Vučetić et al. 2018), and leukocytes (Zhang et al. 2024). In particular, erythrocytes are studied for antibody detection (Wagner and Flegel 1998), analysis of microparticles or macromolecules adhered to the membrane (Grisendi et al. 2015), assessment of erythrocyte shape (Piagnerelli et al. 2007; Gienger et al. 2019), and analysis of erythrocyte osmotic fragility (Yamamoto et al. 2014).

One of the recent studies investigated the biomechanical and biophysical properties of individual red blood cells in sickle cell anemia (SCA), which are considered as potential biomarkers for the diagnosis and monitoring of the disease (Du and Qiang 2026). For this purpose, impedance flow cytometry (IFC) was used as a tool for the non-contact, real-time analysis of individual cells. In a measurement system with integrated hypoxic chambers, IFC allows for measuring the electrical properties (impedance) of single sickle red blood cells under controlled oxygen levels. This approach offers high throughput (up to hundreds of cells per second) and enables the study of cellular population heterogeneity, the detection of sickling kinetics, and the assessment of the impact of therapeutic interventions on cellular properties. This contributes significantly to the development of new diagnostic platforms for SCA. In the study (Palasuwan et al. 2026), a refined flow cytometry method was employed to count malaria-derived particles per microliter of blood. The developed approach is reliable and straightforward for the diagnosis and treatment of patients with malarial parasite infection in routine laboratory settings.

## Integrated assessment of blood microrheology and microcirculation

All methods described above are used to assess parameters of blood microrheology, namely RBC aggregation and deformability. These methods differ from each other in their principles, sensitivity, and measured parameters but most importantly — in the scale of operating (Table 1). For example, optical tweezers operate with single-cell processes, flow chamber allows to study hundreds to thousands of cells, while ektacytometry averages the parameters of millions to billions of cells. This difference is not only in the sample size, the process of RBC aggregation is not the same when we speak about single cells (initial stages of aggregate formation) and cell ensembles (where the initial phase of aggregation is followed by later stages of aggregate-aggregate interaction). This leads to the major advantage of multimodal approach in assessing blood microrheology via biophotonic techniques: combining methods not only gives additional biomarkers of diseases but also allows to gain more complex vision of the process and to look deeper into the mechanisms behind the pathologies. Another significant difference between the methods described is whether the information on studied microrheological parameters is initially obtained as an average over a cell population or from the individual cells separately and averaged only afterwards. For example, in laser aggregometry and RBC diffractometry, such as provided by the RheoScan device, a laser beam approximately 2 mm in width is used; accordingly, the studied parameters are averaged over the entire measurement volume of the cuvette illuminated by the laser, which means data on millions of cells. In contrast, the flow chamber method allows for obtaining deformability parameters from single cells entering the measurement volume. As already described in the corresponding section, this enables not only the measurement of an average value of deformability index but also the cell distributions by deformability and size, which is a significant advantage of this method. This results in a significantly higher sensitivity to anomalous deformability subpopulations of cells by flow chamber compared with ektacytometry. As for the RBC aggregation analysis the techniques listed can be described using the following pattern: pair RBC aggregation (initial phase of aggregation) is studied by optical tweezers in detail, flow chamber provides more general data not specifically on the initial aggregation phase but rather on the later stages of aggregation (more RBCs are included into small aggregates (2–10 cells), these aggregates begin interaction), laser aggregometry operates at a larger scale and provides more detailed data on the last stages of aggregation. We subjectively see the time frames of the mentioned processes as following: initial stage of aggregation (pair of cells) 1–10 s, intermediate stage (formation of small RBC aggregates) 10–60 s, late stage (interaction between huge aggregates containing hundreds of cells) 60 s and more.

**Table 1:** The comparison of laser-optic techniques applied to microrheology studies

Technique name	Operating principles	Provided parameters	Number of cells assessed	Sample type	Parameter distribution measurement is possible	Skills required to operate	Technique cost	Commercially available
Optical tweezers	Optical trapping of cells by laser beams	Pair RBC aggregation and disaggregation forces, RBC elongation indices, various parameters on RBC deformability (Yung’s modulus, etc.) and RBC membrane dynamics	Small. Few dozens of cells in case of RBC aggregation. Dozen to hundreds of cells in case of RBC deformability	Single cells in autologous plasma or polymer solution	Theoretically yes, practically difficult due to small sample size	Very high. Cells are trapped and controlled manually or in a semi-automatized mode	Thousands of USD (5–20)	Yes (Thorlabs, RP photonics, Quantum design, Bruker, etc.)
Flow chamber	Cells are elongated in flow, microscope is used to obtain images	RBC elongation index and its distribution, cell size distribution, RBC aggregation kinetics (Aggregation index, etc.)	Intermediate. Hundreds of cells to thousands of cells	Highly-diluted blood suspension	Yes	Intermediate. Easy to apply automatized measurements and data processing	Few thousand of USD (1–5)	No
Laser aggregometry	Laser beam is scattered on the ensemble of cells. The intensity of scattered light is connected with mean particle size, thus allowing aggregation kinetics assessment	Data on aggregation kinetics. Aggregation index, T1/2, AMP, y0, A1, A2, tau 1, tau 2	Large. Millions to billions of cells	Whole blood	No	Very low. Operating and data processing are automized and the ready-to-use kits are utilized	Dozens of thousands of USD (~ 30)	Yes (LORCA, Rheoscan, Rheodyn SSD, Mizar)
Laser diffractometry (Ektacytometry)	The laser diffraction on cell ensemble pattern is utilized for RBC deformability assessment	Elongation index, yield stress, internal RBC viscosity	Large. Millions of cells	Highly-diluted blood suspension	No	Very low. Operating and data processing are automized and the ready-to-use kits are utilized	Dozens of thousands of USD (~ 30)	Yes (LORCA, Rheoscan, Rheodyn SSD, Mizar)
Flow cytometry	The laser beam is scattered on a stream of single cells. FSC correlates with cell size, SSC correlates with cell internal complexity. Fluorescence signals from single cells can also be detected, enabling multiparametric analysis	Relative cell size, relative granularity, morphological parameters (cell shape, diameter, area, aspect ratio etc.), fluorescence-based parameters (amount of the protein/fluorophore molecules in individual cells)	Hundreds of cells to thousands of cells	Single cells	Yes	Intermediate. Operating process is automized, however, data processing may be challenging	From dozens of thousands of USD (~ 90) to million USD	Yes (Thermo Fisher Scientific, Miltenyi Biotec, BD Biosciences, etc.)

Despite the mentioned drawbacks, the laser aggregometry and diffractometry methods hold an important advantage over other methods. For these methods, various commercial instruments exist, equipped with designed experimental protocols, ready-to-use consumables, and software (Table 1). This significantly speeds up its usage and minimizes errors arising from sample preparation, cuvette fabrication, and human factors during experimentation, which can occur in the flow chamber and optical tweezers methods.

## Conclusion

In this brief review, we provide an up-to-date description of five experimental techniques widely used to assess microcirculatory parameters of human blood, namely the parameters of RBC deformability and aggregation. The descriptions of working principles of the methods are accompanied by novel results and possible approaches. The advantages and limitations are discussed for each method reviewed. The limitations of our review itself are the following: we did not discuss numerical approaches applied to estimate RBC aggregation and deformability (for example, (Sigüenza et al. 2017; Geekiyanage et al. 2020)), we also did not review in detail such experimental techniques as atomic force microscopy, scanning electron microscopy, etc. For detailed information on these methods and its applications in the field of microcirculation studies see, for example, (Adan et al. 2017; Yeow et al. 2017; Krieg et al. 2018; Smith and Starborg 2019). We hope our review would be useful both for beginner researchers in the field of microcirculation and for senior scientists interested in applying new experimental techniques.

## Data Availability

No datasets were generated or analysed during the current study.
